# The lupus band test: A review of the sensitivity and specificity in the diagnosis of lupus erythematosus

**DOI:** 10.1002/ski2.205

**Published:** 2023-07-11

**Authors:** Sarah Ní Maolcatha, Ellis Nic Dhonncha, Cathal O’Connor, Sinead Dinneen, Cynthia C. B. B. Heffron

**Affiliations:** ^1^ Pathology Cork University Hospital Cork Ireland; ^2^ Dermatology South Infirmary Victoria University Hospital Cork Ireland; ^3^ Paediatrics and Child Health University College Cork Cork Ireland; ^4^ INFANT Research Centre University College Cork Cork Ireland

## Abstract

**Background:**

The lupus band test (LBT) is a direct immunofluorescence (DIF) technique which shows a band of localised immunoglobulins at the dermo‐epidermal junction in lesional, non‐sun‐exposed skin of patients with both systemic and cutaneous lupus erythematosus (LE), and in perilesional skin of patients with systemic LE. However, low sensitivity and poor concordance between histological and clinical diagnoses warrant a review of the application of the LBT in the diagnosis of LE.

**Objectives:**

To assess the sensitivity and specificity of the LBT in diagnosing LE following clinico‐pathological correlation (CPC).

**Methods:**

All cases sent to our pathology department between 2011 and 2018 for DIF with a clinical query of LE were reviewed. Data collection included demographic details, pathology requests, histology and DIF reports, clinical reports and diagnoses, and serology.

**Results:**

Of 256 histology requests, 9% (*n* = 23) had a positive LBT. This was discordant with the prevalence of LE diagnosis, as 46.3% were diagnosed with LE following CPC. The sensitivity and specificity of the LBT for LE was 17.6% and 98.8% respectively, with a positive predictive value of 92.9% and negative predictive value of 58.2%.

**Conclusion:**

The LBT is not a sensitive diagnostic test for LE, but is highly specific, and should be considered as a supportive diagnostic tool for LE. This is the largest reported case series evaluating the efficacy of the LBT in the diagnosis of LE.

## Graphical abstract

The lupus band test (LBT) is a direct immunofluorescence (DIF) technique which shows a band of localised immunoglobulins at the dermo‐epidermal junction in lesional, non‐sun‐exposed skin of patients with both systemic and cutaneous lupus erythematosus (LE). In this large review, the sensitivity and specificity of the LBT for LE was 17.6% and 98.8% respectively, with a positive predictive value of 92.9% and negative predictive value of 58.2%. The LBT is not a sensitive diagnostic test for LE, but is highly specific, and should be considered as a supportive diagnostic tool for LE.

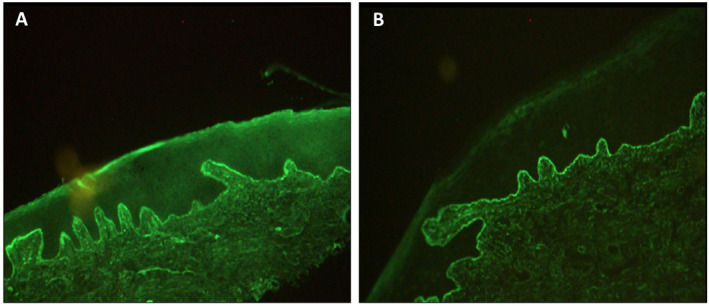

1

**What is already known about this topic?**

- The lupus band test (LBT) is a direct immunofluorescence technique which shows a band of localised immunoglobulins at the dermo‐epidermal junction in lesional, non‐sun‐exposed skin of patients with both systemic and cutaneous lupus erythematosus (LE), and in perilesional skin of patients with systemic LE. However, low sensitivity and poor concordance between histological and clinical diagnoses warrant a review of the application of the LBT in the diagnosis of LE.

**What does this study add?**

- This review of 256 cases showed that the sensitivity and specificity of the LBT for LE was 17.6% and 98.8% respectively, with a positive predictive value of 92.9% and negative predictive value of 58.2%. The LBT is not a sensitive diagnostic test for LE, but is highly specific, and should be considered as a supportive diagnostic tool for LE. This is the largest reported case series evaluating the efficacy of the LBT in the diagnosis of LE.

## 2 INTRODUCTION

Skin is the second most common organ involved in systemic lupus erythematosus (SLE), and is the only organ affected in cutaneous lupus erythematosus (CLE).^1^ In SLE, 15% of patients present with cutaneous manifestations,^2^ and 85% of patients with SLE will have a cutaneous manifestation of lupus erythematosus (LE) at some point, most commonly photo‐distributed erythema.^3^ The diagnosis of SLE is made on clinical, histopathological and serological findings. The histological features of the subsets of CLE overlap and vary with severity of disease, making clinico‐pathological correlation (CPC) essential. Serological evaluation includes antinuclear antibodies (ANA), anti‐double‐stranded DNA (dsDNA), anti‐Smith (Sm), and anti‐Ro/SS‐A and anti‐La/SS‐B antibodies.^4^

A further diagnostic tool is the lupus band test (LBT), a fluorescent band of immunoglobulins (IgG, IgM and IgA) demonstrated at the dermo‐epidermal junction (DEJ) in patients with LE, which can be homogenous, thready or stippled.^5^, ^6^ The LBT is positive in lesional skin of 50%–94% of patients with SLE, 60% of patients with subacute CLE (SCLE) and 60–80% of patients with discoid LE (DLE).^7^, ^8^, ^9^ A positive LBT has been reported in perilesional skin of 67% of patients with SLE.^10^ There is a reported higher rate of positive LBTs in sun‐exposed skin (60%–70%) compared to sun‐protected skin (50%–60%).^7^ Furthermore, 20% of biopsies of sun‐exposed normal skin of normal young adults may also have a positive LBT.^11^ It is hypothesised that denaturation of keratinocyte DNA by ultraviolet light crosses the basal membrane (BM) and becomes trapped, is bound to circulating ANA, and forms granular deposits at the BM.^12^, ^13^

To support the diagnosis of LE, direct immunofluorescence (DIF) may be considered in addition to histology at initial assessment.^14^, ^15^ However, low sensitivity and poor concordance with histological and clinical diagnoses have warranted a critical review of the utility of the LBT in the diagnosis of LE. The aim of this study was to assess the sensitivity and specificity of the LBT in diagnosing LE following CPC.

## 3 METHODS

All skin biopsies submitted to our department for DIF between 2011 and 2018 were analysed. All request forms received for DIF were reviewed and key data recorded in a database: sex; age; clinical differential diagnosis; location of biopsy site, and type of skin at biopsy site (lesional, perilesional or normal skin). The final reports of all cases were reviewed and key characteristics recorded: lupus band test result (positive/negative); nature of immune deposits (IgG; IgM; IgA; C3); pattern of immune deposits and intensity of fluorescence (+1, +2 or +3). All biopsies reported as being positive for the LBT had a linear band deposition of immunoglobulins at the BM. The histology report of the corresponding skin biopsies were reviewed and their data captured in the database: type of skin at biopsy site (lesional or perilesional); and final diagnosis on histology. Histology reports were available for all cases.

Serological laboratory results were reviewed from electronic hospital databases, for analysis of ANA and other LE‐related antibodies.

Written and electronic clinical records were reviewed for CPC and to ascertain if patients were definitively diagnosed and treated for LE following CPC. CPC involved review by the dermatology and pathology team, and other relevant teams, classifying SLE based on the European League Against Rheumatism/American College of Rheumatology criteria,^16^, ^17^ and diagnosing CLE based on clinical and histological features.

### 3.1 Statistical methods

Using Microsoft Excel and SPSS, sensitivity and specificity were calculated for the LBT, histology, and serology using diagnosis following CPC as the gold standard of diagnosis. In calculating the sensitivity and specificity, only those cases with a recorded clinical diagnosis were used. The sensitivity and specificity were calculated using the conventional two by two table.

## 4 RESULTS

Of 947 skin biopsies received for DIF, 27% (256/947) had a clinical query that included LE (Table 1). Of these, in 43.4% (111/256) LE was the main suspected diagnosis, while 56.6% (145/256) of requests included LE in a list of multiple differential diagnoses. The mean age of patients with a suspicion of LE was 55 years and 75% were female. Sun‐exposed sites including the face, neck and hand accounted for 39.8% (102/256) of all DIF biopsies received for review and 52.2% (12/23) of biopsies with a positive LBT. DIF biopsies were labelled as lesional in 31.6% (*n* = 81) of samples, non‐lesional in 11.3% (*n* = 29), and 57% (*n* = 146) were not specified.

**TABLE 1:** Patient demographics and biopsy characteristics for LBT (*n* = 256)

Mean age (years)	55
Gender	
Female *n* (%)	191 (74.6%)
Male *n* (%)	65 (25.4%)
Location of biopsy site	
Head and neck	94 (36.7%)
Upper limb	77 (30.1%)
Chest	57 (22.3%)
Lower limb	26 (10.2%)
Not documented	2 (0.8%)
Type of skin	
Lesional	81 (31.6%)
Non‐lesional	29 (11.3%)
Unknown	146 (57%)

A positive LBT was seen in 9% (23/256) of all biopsies for DIF (Table 2). LE was diagnosed in 39.8% (102/256) of skin biopsies on histology, of which 18.6% (19/102) had a positive LBT. Only 37% (95/256) of patients had serology tested for autoantibodies (Table 2). All 95 samples were tested for ANA, of which 60% (*n* = 57) were positive. LE‐specific antibodies (dsDNA, anti‐Sm, Ro/SS‐A, La/SS‐B) were tested in 48.4% (*n* = 46) of those who had ANA testing. Positive dsDNA was seen in 5.3% (5/95), positive anti‐Sm in 3.2% (3/95), positive Ro/SS‐A in 31.6% (30/95) and positive La/SS‐B in 17.9% (17/95).

**TABLE 2:** Results of histology, direct immunofluorescence, serology, and clinical diagnosis following clinico‐pathological correlation

Direct immunofluorescence (*n* = 256)	
Positive LBT	23 (9%)
Negative LBT	233 (91%)
Histological diagnosis (*n* = 256)	
Lupus erythematosus	102 (39.8%)
Other	154 (60.2%)
Serology (*n* = 95)	
Positive ANA	57/95 (60%)
Positive dsDNA and/or Anti‐Sm	6/46 (13%)
Positive anti Ro/SS‐A/Anti La/SS‐B	30/46 (65.2%)
Clinical diagnosis (*n* = 160)	
Lupus erythematosus	74 (46.3%)
Psoriasis	7 (4.4%)
Eczema	5 (3%)
Other	74 (46.3%)

Abbreviations: ANA, antinuclear antibody; Anti Ro/SS‐A, Anti‐Sjögren's‐syndrome related antigen A; Anti‐Sm, Anti‐Smith; dsDNA, double‐stranded deoxyribonucleic acid; La/SS‐B, Anti‐Sjögren's‐syndrome related antigen B; LBT, lupus band test.

A definitive diagnosis following CPC was documented on 62.5% (160/256) of cases. LE was diagnosed in 74 of the 160 patients (46.3%).

LE was diagnosed in 39.8% (102/256) of skin biopsies on histology, of which 18.6% (19/102) had a positive LBT. A positive LBT was seen in 9% (23/256) of all biopsies for DIF (Table 2). Details of the clinical management of these patients were available for 60.9% (14/23) of cases, with 92.9% (13/14) managed clinically for LE (Table 3). Histological features were consistent with LE in 82.6% (19/23) of those with a positive LBT. Autoantibody testing was performed in 12 of those with a positive LBT, of which 83.3% (10/12) were positive for ANA alone or in association with dsDNA, Ro/SS‐A, and La/SS‐B. Overall, 87% (20/23) of cases with a positive LBT had either one, two or three of the following: clinical diagnosis of LE, histology consistent with LE, or serology consistent with LE.

**TABLE 3:** Histological, serological and clinical diagnosis of all cases with a positive lupus band test

Case	Site	Type	Histological diagnosis	Serology	Clinical diagnosis
1	Face	Non‐lesional	Actinic keratosis	Not tested	NA
2	Neck	Lesional	Dermatitis	Not tested	NA
3	Arm	Unknown	LE	ANA, Ro/SS‐A, La/SS‐B	NA
4	Ear	Unknown	LE	ANA^a^	NA
5	Leg	Lesional	Necrobiosis lipoidica	Not tested	NA
6	Face	Unknown	LE	Not tested	NA
7	Arm	Non‐lesional	LE	ANA, Ro/SS‐A, La/SS‐B	LE
8	Hand	Unknown	LE	ANA, Ro/SS‐A	LE
9	Neck	Unknown	LE	ANA, dsDNA^a^	LE
10	Neck	Unknown	LE	Not tested	LE
11	Arm	Unknown	Dermatitis	ANA, dsDNA	LE
12	Arm	Lesional	LE	ANA, dsDNA, Ro/SS‐A, La/SS‐B	NA
13	Arm	Lesional	LE	ANA, dsDNA, Ro/SS‐A,	NA
14	Chest	Non‐lesional	LE	Not tested	LE
15	Chest	Unknown	LE	ANA^b^	LE
16	Forehead	Lesional	LE	Not tested	LE
17	Arm	Lesional	LE	Not tested	LE
18	Arm	Non‐lesional	LE	Not tested	LE
19	Face	Unknown	LE	ANA^b^	NA
20	Face	Lesional	LE	Not tested	LE
21	Arm	Unknown	LE	ANA	LE
22	Face	Unknown	LE	Not tested	LE
23	Face	Unknown	LE	ANA	NA

Abbreviations: ANA, antinuclear antibody; Anti Ro/SS‐A, Anti‐Sjögren's‐syndrome related antigen A; Anti‐Sm, Anti‐Smith; dsDNA, double‐stranded deoxyribonucleic acid; La/SS‐B, Anti‐Sjögren's‐syndrome related antigen B; LE, Lupus Erythematosus; NA, not available.

^a^ Tested for ANA and antibodies to dsDNA only.

^b^ Tested for ANA only.

**FIGURE 1:**
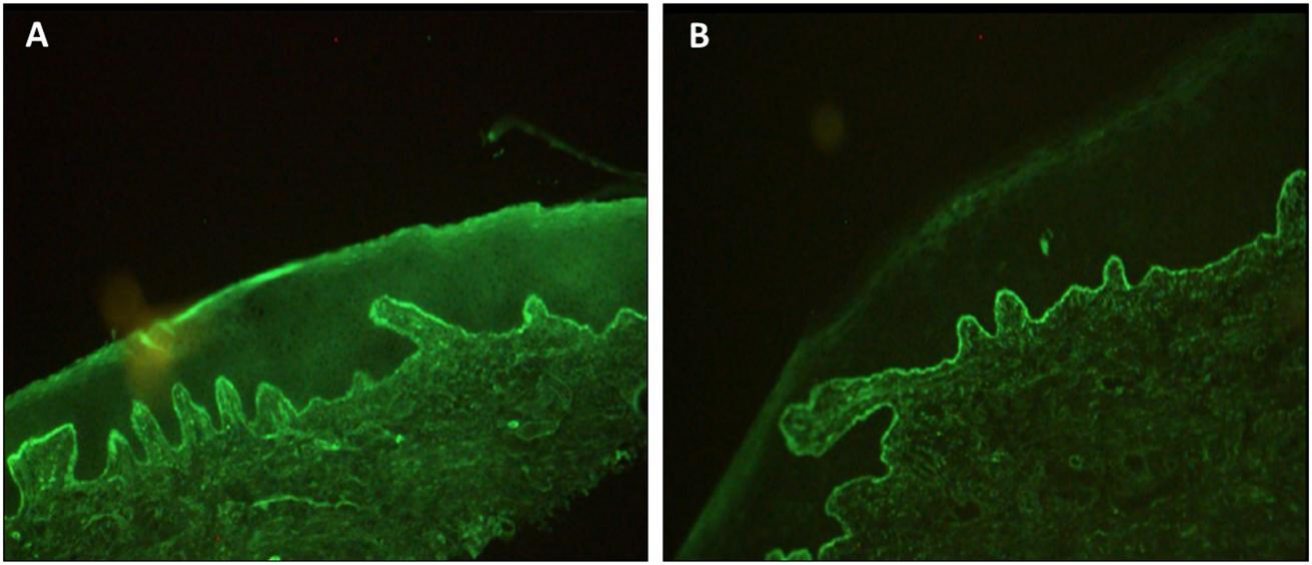
Direct immunofluorescence showing granular linear band of IgG (a) and IgM (b) along the dermo‐epidermal junction

**FIGURE 2:**
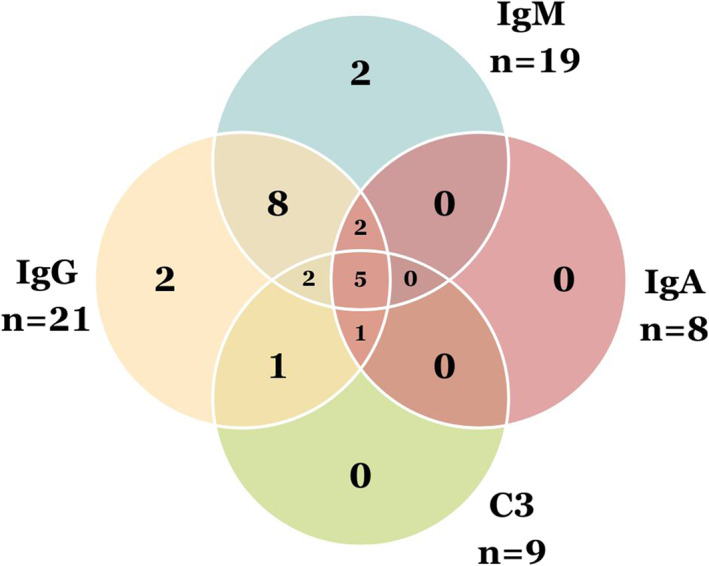
Direct immunofluorescence, frequency of Immunoglobulins and complement at basement membrane in positive LBT cases. C3, Complement C3

In all cases of LBT positivity a homogenous thready or granular band of immunoglobulins and complement was present at the BM (Figure 1). IgG was positive in 91.3% (21/23) and IgM was positive in 82.6% (19/23) of cases with a positive LBT (Figure 2). Complement 3 (C3) was seen in 39.1% (9/23) and always with IgG or IgM. IgA was seen in 34.8% (8/23) and always with IgG or IgM. The intensity of antibody staining ranged from +1 to +2.

Overall, 91% (233/256) of samples for DIF with a clinical suspicion of LE had a negative LBT. Of the cases with a negative LBT, 7% (17/233) had serological, clinical, and histological features consistent with LE. Overall, 46.3% (109/233) with a negative LBT had either one, two or three of the following: clinical diagnosis of LE, histology consistent with LE, serology consistent with LE.

The sensitivity and specificity of LBT for diagnosing LE were 17.6% and 98.8% respectively, giving a positive predictive value (PPV) of 92.9% and negative predictive value (NPV) of 58.2% (Table 4). The sensitivity and specificity of histology alone for diagnosing LE were 86.5% and 90.7% respectively, giving a positive predictive value of 88.9% and negative predictive value of 88.6%.

**TABLE 4:** Sensitivity, Specificity, Positive Predictive Value and Negative Predictive Value of the LBT and Histology in the diagnosis of LE

	LBT (*n* = 160)	Histology (*n* = 160)
True positive	13	64
True negative	85	78
False positive	1	8
False negative	61	10
Sensitivity	17.6%	86.5%
Specificity	98.8%	90.7%
Positive predictive value	92.9%	88.9%
Negative predictive value	58.2%	88.6%

Abbreviation: LBT, lupus band test.

## 5 DISCUSSION

The diagnostic algorithm of LE is relatively complex, involving clinical, serological, and histological correlation to diagnosis and subclassify the disease. Misdiagnosis or delayed diagnosis of LE can lead to serious complications. Adjunctive diagnostic tools like the LBT can be helpful in particular clinical scenarios, where other investigations are negative or inconsistent. This study examined the application of the LBT in supporting or undermining a diagnosis of LE. It showed that the LBT has high specificity, meaning that a positive test is very likely representative of LE, but low sensitivity, meaning that a negative test does not outrule LE.

A defined and reproducible definition of the LBT has been difficult to substantiate. In 1995 it was defined as the presence of a bright green‐yellow band at the DEJ regardless of the specific constituents.^18^ A subsequent review suggested that an uninterrupted band of IgM involving at least 50% of the basement membrane of sun‐exposed lesional skin should be accepted at initial diagnosis as a positive LBT.^2^ However the most commonly identified immunoglobulin in lesional skin is IgG,^14^, ^19^, ^20^ followed by IgM and IgA.^8^ Most (91.3%) cases in our cohort with a positive LBT had IgG depositions at the basement membrane alone (8.7%) or in conjunction with other immunoglobulins or complement (82.6%). The frequency of IgM deposition in isolation or in combination with other immunoglobulins or complement was 82.7%. Deposits of IgM alone were identified at the basement membrane in one case with a positive LBT, however the reciprocal histological and clinical reviews reported a diagnosis of necrobiosis lipoidica, which has been previously reported.^21^ IgM deposition at the basement membrane can also be found in actinic keratoses, dermatomyositis and polymorphous light eruption as a result of UV exposure.^10^, ^22^, ^23^ For sun‐protected skin, an interrupted band of IgM deposits at the basement membrane of sun of at least moderate intensity is now accepted as a positive LBT.^2^ The presence of multiple immunoreactants, in particular complement, at the basement membrane in a positive LBT, especially a large amount, has been associated with active SLE,^22^, ^23^ although more recent studies have not shown a correlation between type or pattern of immunoreactants and disease activity.^24^

The high specificity (90.7%), sensitivity (86.5%) PPV (88.9%) and NPV (88.6%) of histology alone highlight the strength of this modality in diagnosing LE in skin. The high NPV of the histopathological diagnosis, compared to the LBT (NPV 58.2%), may be also useful in excluding other diseases with a similar clinical presentation to LE. Moreover, on review of the cases that had a negative LBT, 46.2% (61/132) of those with clinical details available were treated and diagnosed clinically with LE. Importantly, 85% (52/61) of those treated clinically with LE had corresponding confirmatory histology for LE. The specificity and sensitivity values reported in this study must be considered in the context of clinical review by experienced dermatologists. If a dermatologist has deemed it necessary to biopsy to investigate for lupus, then the pre‐test probability will be raised as the dermatologist's suspicion of a diagnosis of lupus suggests that other diagnoses are less likely. This may have implications for interpretation of the sensitivity and specificity calculation outside of this clinical context.

A review of published studies looking at the sensitivity and specificity of the LBT shows the wide range of reported figures, clinical variables and criteria used. George et al., examining the LBT in diagnosing DLE (*n* = 28) and SLE (*n* = 32), reported the sensitivity and specificity of the LBT in DLE to be 58% and 87% respectively, and the sensitivity and specificity of the LBT in SLE to be 93% and 87% respectively.^18^ Mysorekar et al. reported 100% LBT positivity in 9 cases of LE from biopsies of lesional skin.^25^ Cardinali et al., using strict criteria of at least two immunoglobulins at the DEJ of sun‐protected non‐lesional skin on patients with known SLE, calculated sensitivity and specificity of 10.5% and 97.8% respectively.^26^ Using a definition of one immunoreactant, sensitivity and specificity of 78.9% and 47.8% were calculated. A recent study reported sensitivity and specificity of 56.5% and 88.2% in non‐lesional skin of patients with SLE (*n* = 57), with minimal difference between sun‐protected and sun‐exposed skin.^24^ The retrospective nature of our study must be taken into account in interpreting our sensitivity and specificity results, as not all variables were always available for analysis for example, biopsy site, previous treatment, duration of lesions, and clinical certainty of LE. A retrospective review of DIF and histology of DLE cases found that these variables were also likely to have compounded their results of LBT positivity in 68% of their tested cohort.^20^

Strengths of this study include the large size of the cohort; the triangulation of clinical, histological, and serological data for CPC; and the assessment of all DIF samples over a long time period. Limitations of the study include the single‐centre nature; the retrospective data capture, which made it difficult to access some datapoints; the low rates of biopsy site documentation and serology assessment; and the lack of data on treatment or disease activity. Duration of the lesion and treatment may affect immune deposits and therefore contribute further to false negatives.^27^, ^28^

On review of our study and other previous studies incorporating all the variables, the LBT should be considered of most helpful additional diagnostic value in ruling in lupus if other parameters are negative or conflicting and there is a high clinical suspicion of LE, as with a specificity of 98.84% and a PPV of 92.86%, false positives are highly unlikely. We suggest that if a DIF is being considered as part of LE workup, a lesional, sun‐exposed site should initially be biopsied to increase sensitivity. Additional perilesional biopsies can be considered to help in subclassifying between SLE and CLE.

## 6 CONCLUSION

This is the largest reported case series evaluating the utility of the LBT in the diagnosis of LE and reflects clinical practice in the region. Over one quarter of our biopsy samples received for DIF were for assessment for LE. The use of the LBT is complicated by the variation in LBT positivity across previous studies, the multitude of compounding variables, and the importance of clinico‐immuno‐histopathological correlation in the diagnosis of LE. Interpretation of the LBT requires awareness of sun‐exposure at the biopsy site, disease activity and treatment, and clinical, serological and histological correlation. Given the low sensitivity but very high specificity, it may be more prudent and cost‐effective to use DIF as an ancillary expert diagnostic tool to ‘rule in’ LE in diagnostically challenging cases, as opposed to ‘rule out’ LE.

## CONFLICTS OF INTEREST

The authors declare that there is no conflict of interest.

## AUTHOR CONTRIBUTIONS

**Sarah Ní Maolcatha**: Conceptualization (Equal); Data curation (Equal); Formal analysis (Equal); Funding acquisition (Equal); Investigation (Equal); Methodology (Equal); Project administration (Equal); Resources (Equal); Software (Equal); Supervision (Equal); Validation (Equal); Visualization (Equal); Writing – original draft (Equal); Writing – review & editing (Equal). **Ellis Nic Dhonncha**: Data curation (Equal); Formal analysis (Equal); Investigation (Equal); Methodology (Equal); Writing – review & editing (Equal). **Cathal O’Connor**: Data curation (Equal); Formal analysis (Equal); Investigation (Equal); Methodology (Equal); Project administration (Equal); Resources (Equal); Software (Equal); Supervision (Equal); Validation (Equal); Visualization (Equal); Writing – review & editing (Equal). **Sinead Dinneen**: Data curation (Equal); Formal analysis (Equal); Investigation (Equal); Methodology (Equal); Resources (Equal); Software (Equal); Writing – review & editing (Equal). **Cynthia C. B. B. Heffron**: Conceptualization (Equal); Data curation (Equal); Formal analysis (Equal); Funding acquisition (Equal); Investigation (Equal); Methodology (Equal); Project administration (Equal); Resources (Equal); Software (Equal); Supervision (Equal); Validation (Equal); Visualization (Equal); Writing – original draft (Equal); Writing – review & editing (Equal).

## ETHICS STATEMENT

Ethical approval was obtained from the Clinical Research Ethics Committee of the Cork Teaching Hospitals.

## Data Availability

The data that support the findings of this study are available from the corresponding author upon reasonable request.
